# Supplementary material for: An atlas of the binding specificities of transcription factors in Pseudomonas aeruginosa directs prediction of novel regulators in virulence
Source: eLife. 2021 Mar 29;10:e61885. doi: 10.7554/eLife.61885 (PMC8041468; doi:10.7554/eLife.61885)
Supplement: Supplementary file 3. — Diamonds indicate TFs, and circles indicate individual PWMs. The dashed lines show the motif of the TF. The TFs without names are named with their locus tag omitting ‘PA’, related to Figure 1B. [file elife-61885-supp3.pdf]

Supplementary File 3

The 104 distinct modules of the obtained 198 PWMs for 182 TFs. Diamonds indicate TFs, circles indicate individual PWMs. The dashed lines show the motif of the TF. The TFs without names are named with their locus tag omitting “PA”.

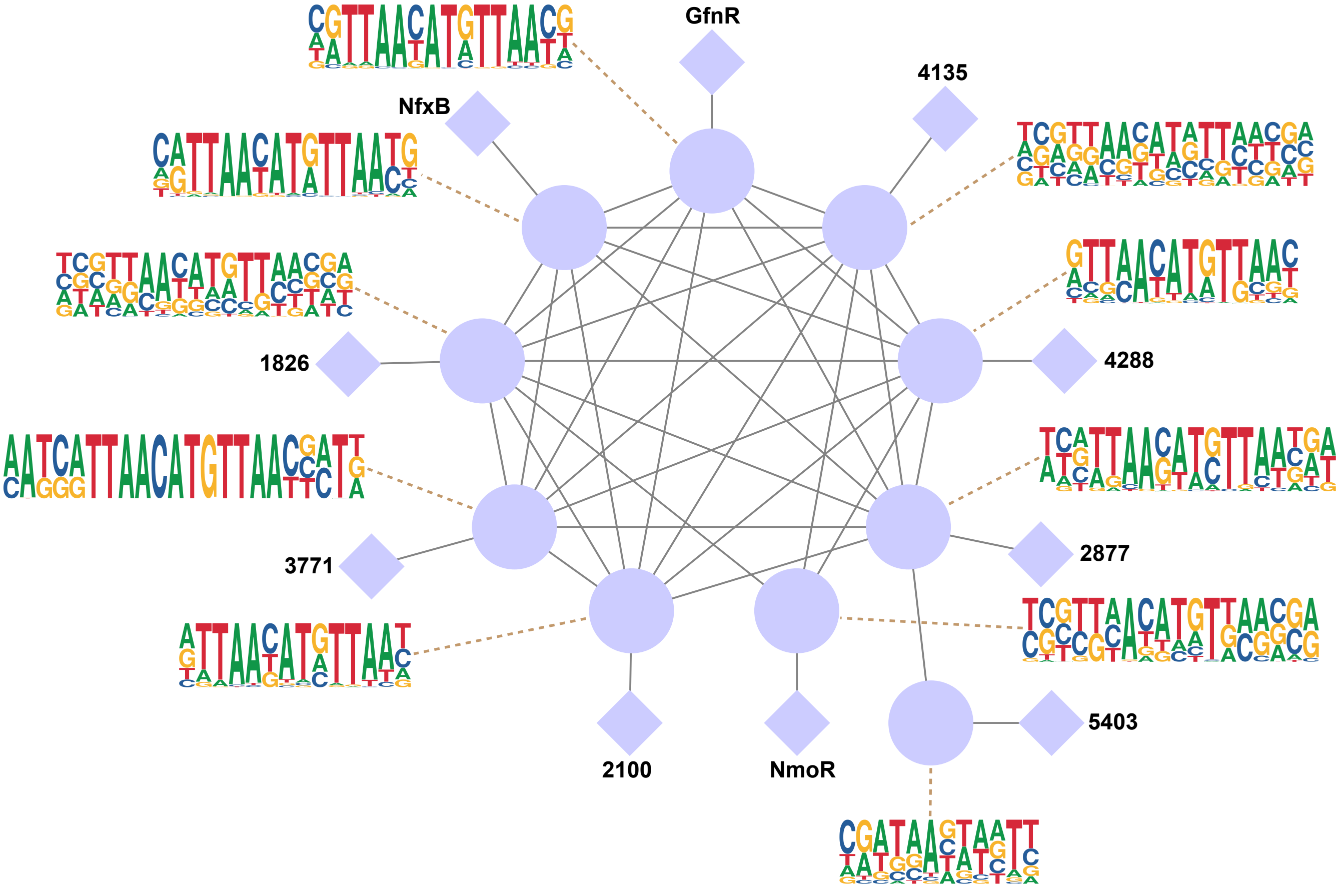

Module 1

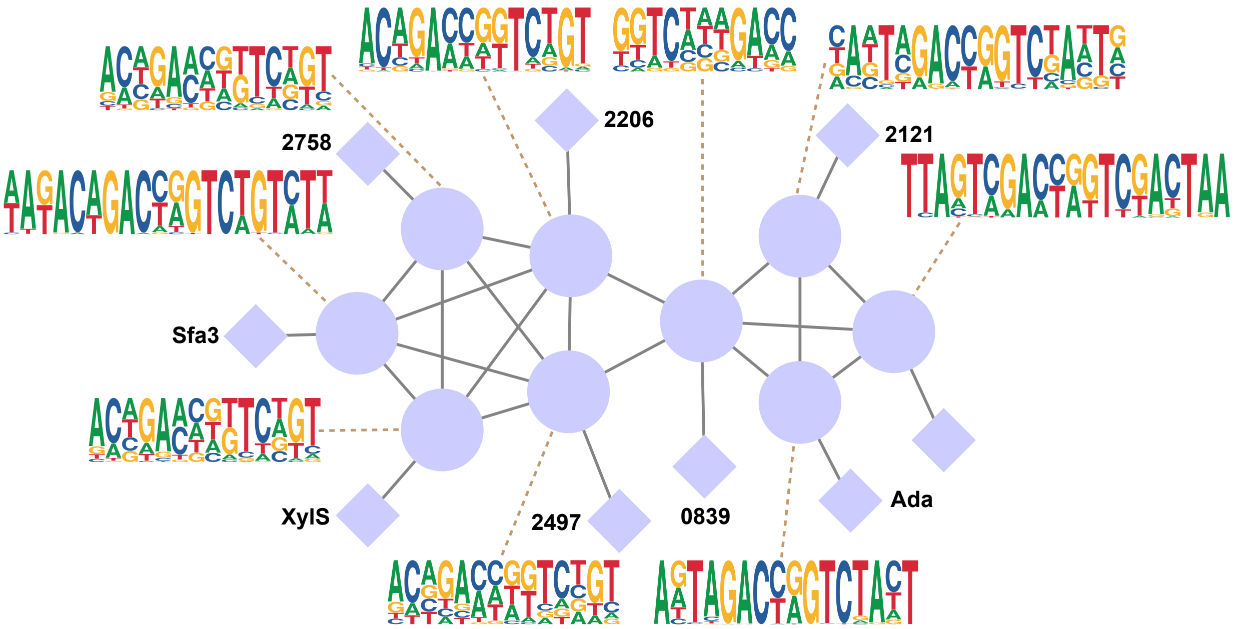

Module 2

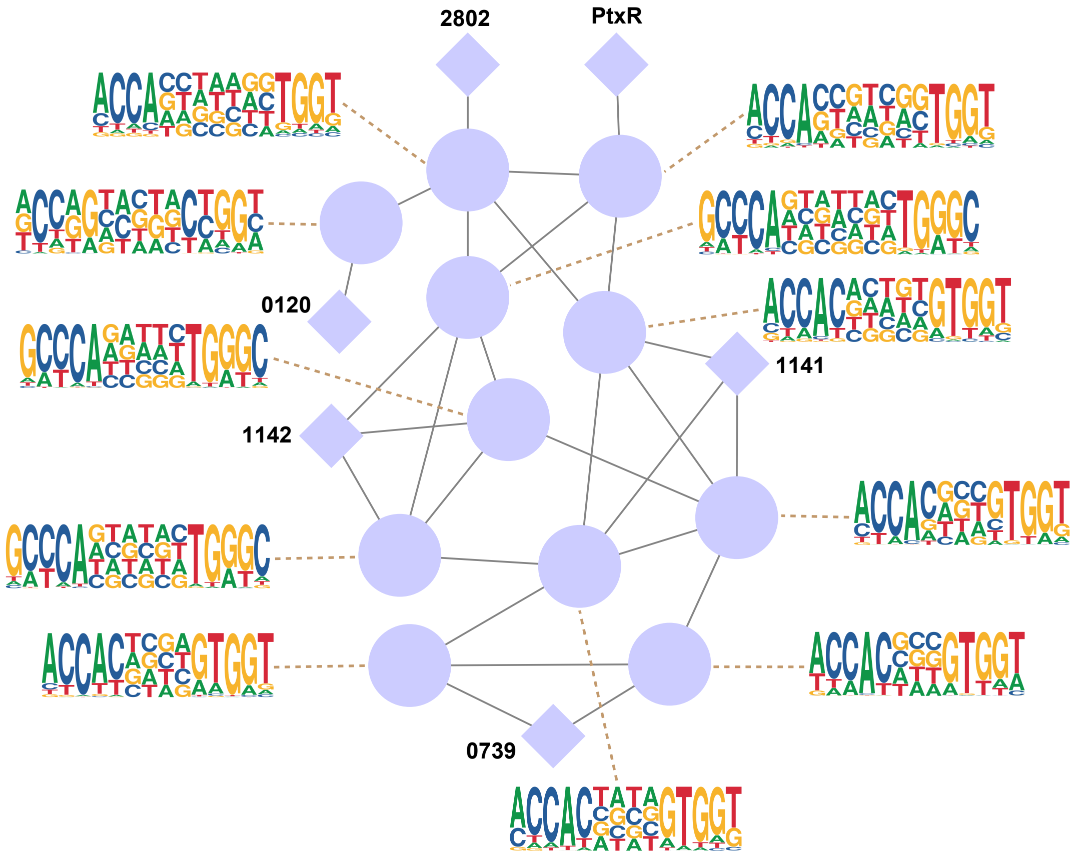

Module 3

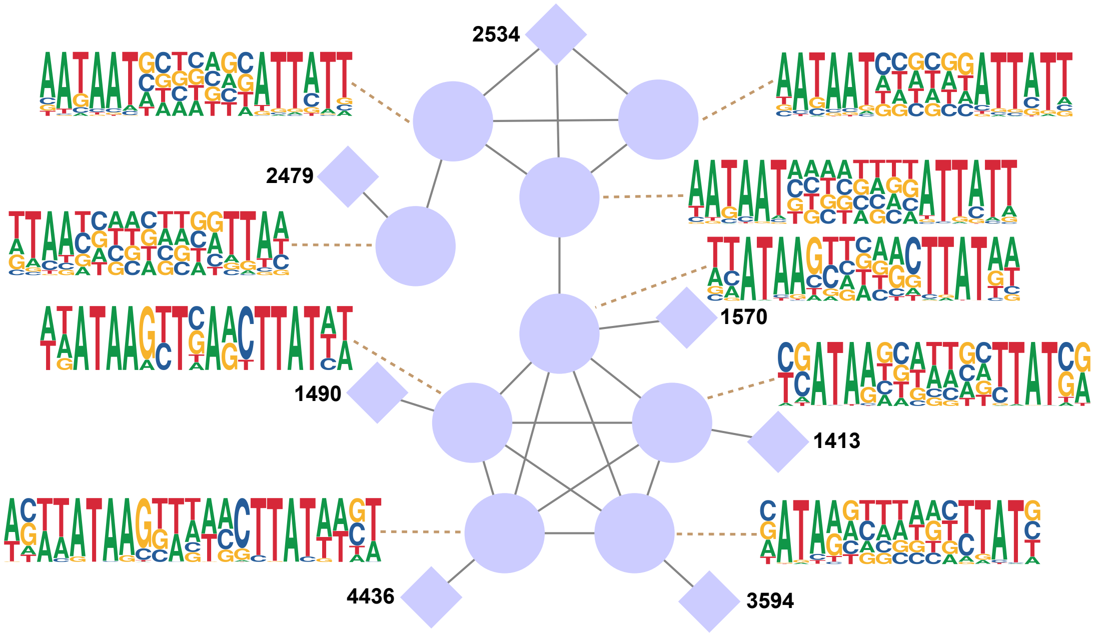

Module 4

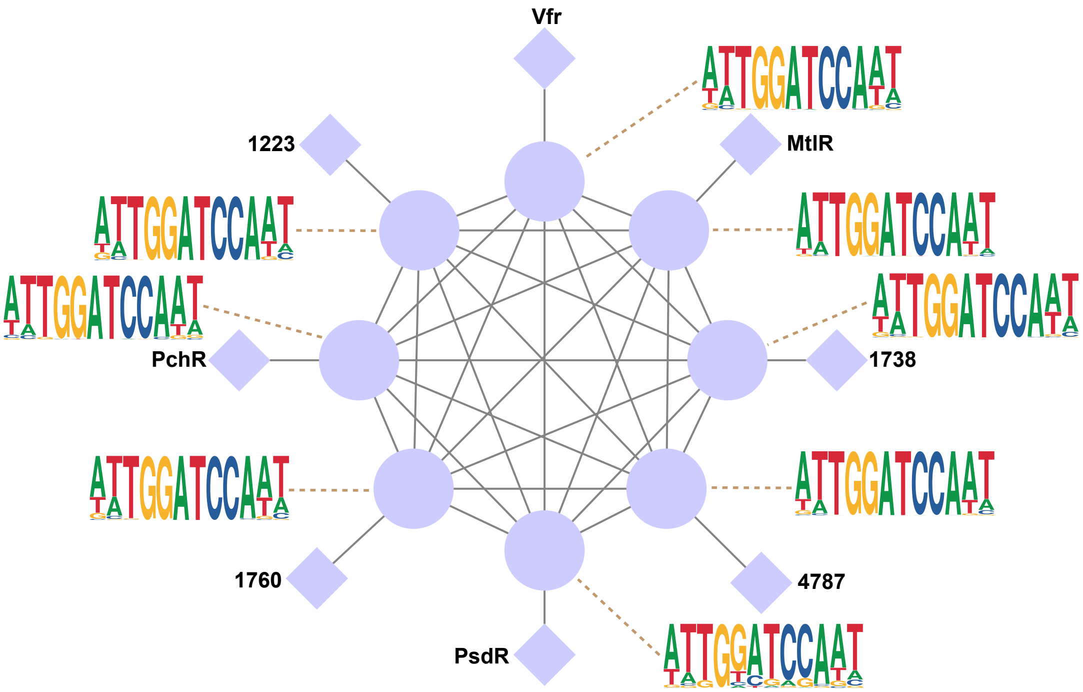

Module 5

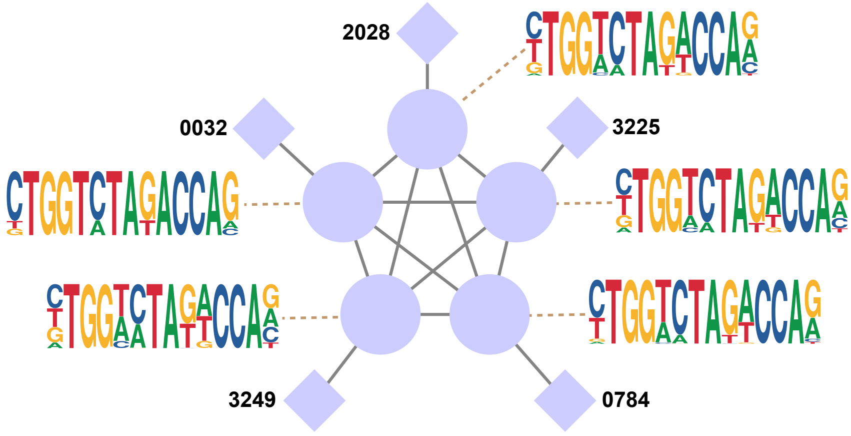

Module 6

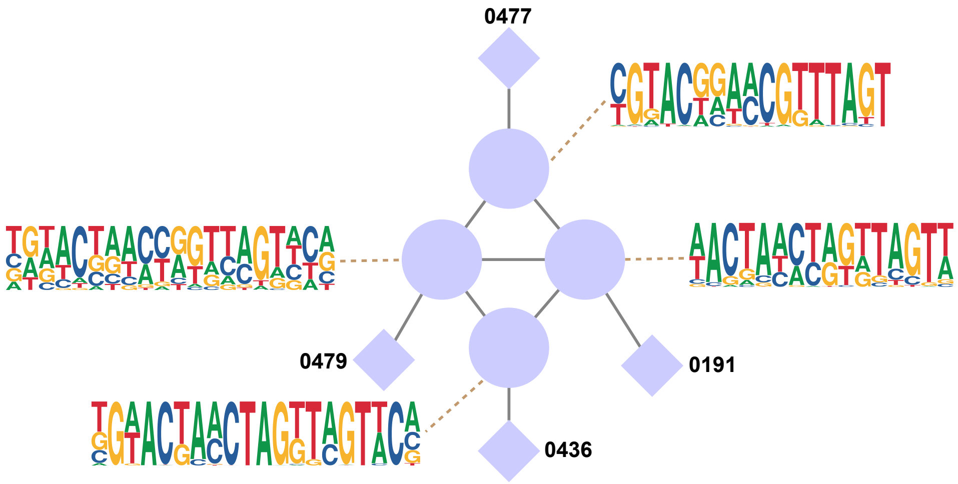

Module 7

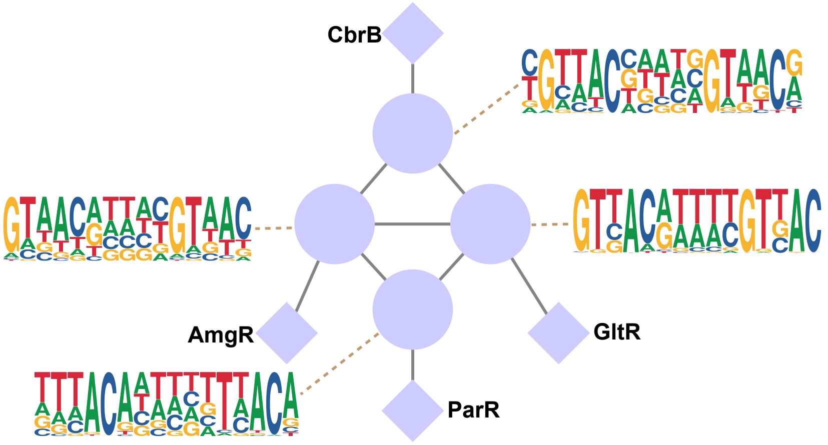

Module 8

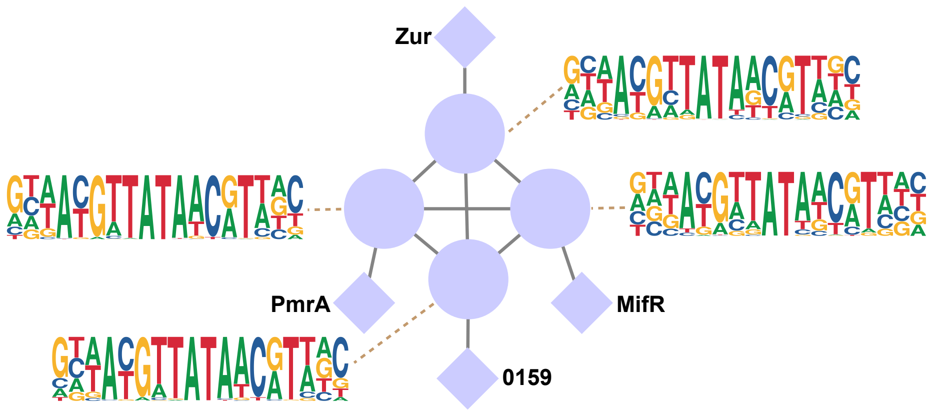

Module 9

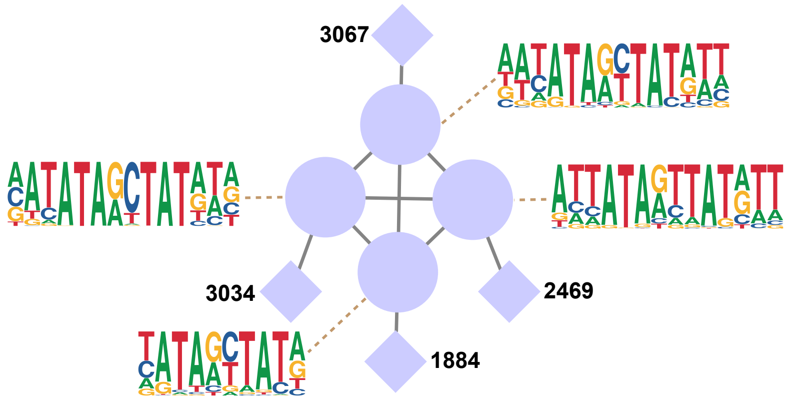

Module 10

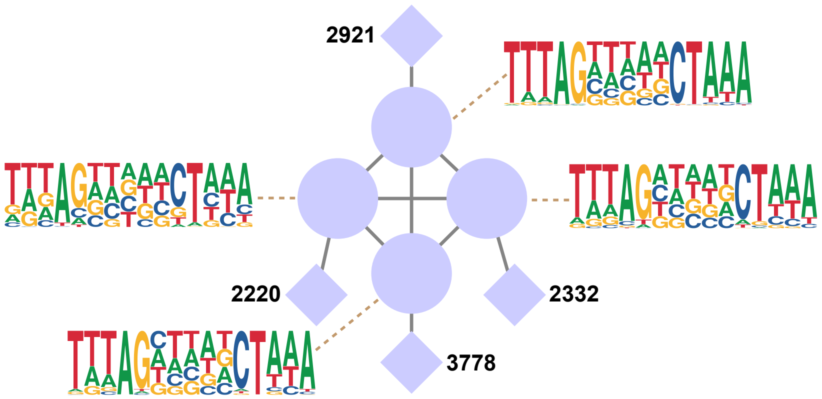

Module 11

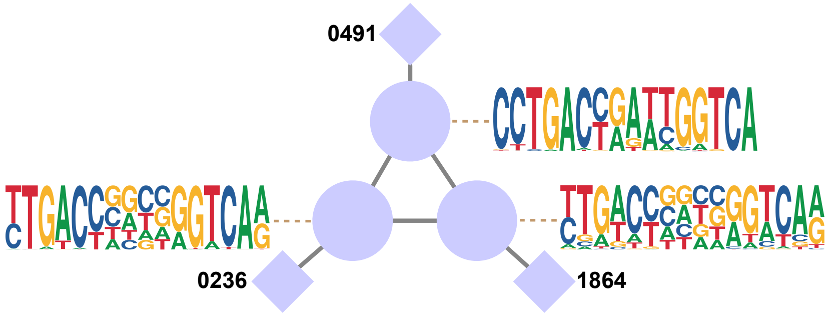

Module 12

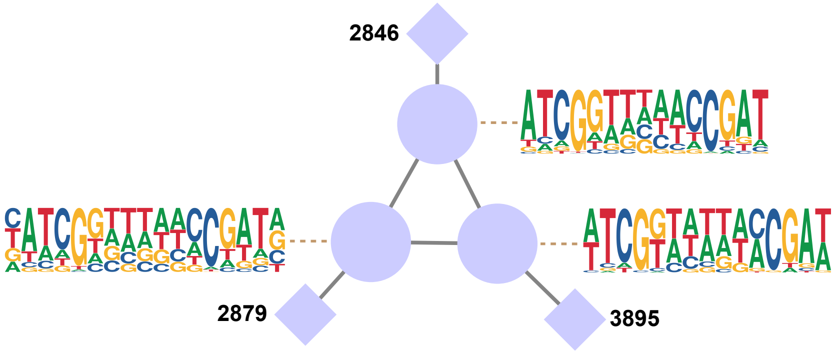

Module 13

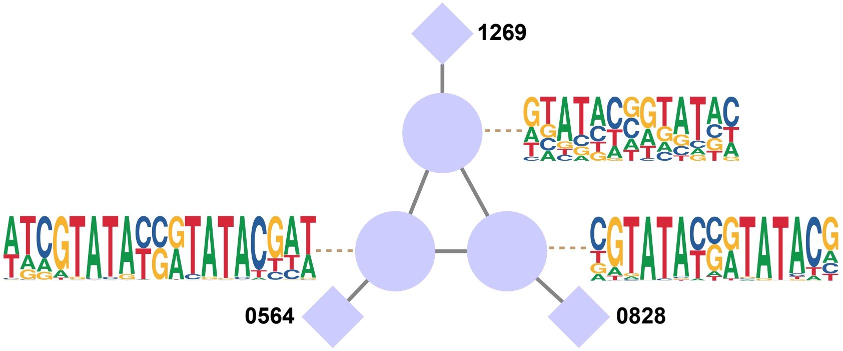

Module 14

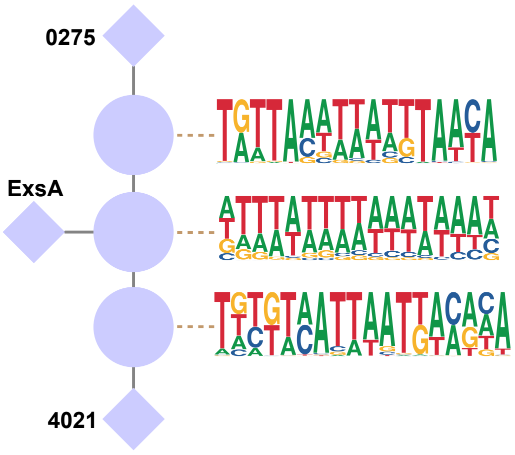

Module 15

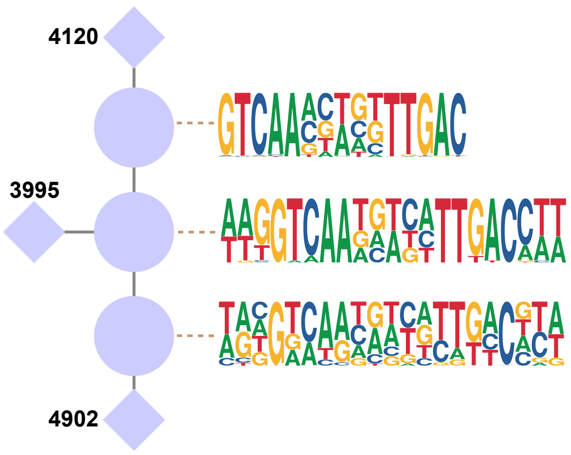

Module 16

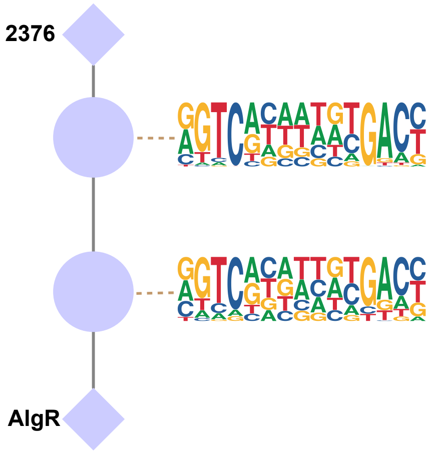

Module 17

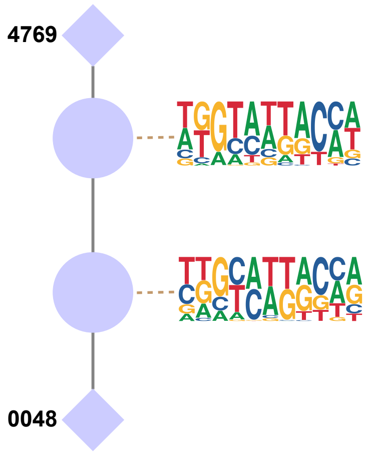

Module 18

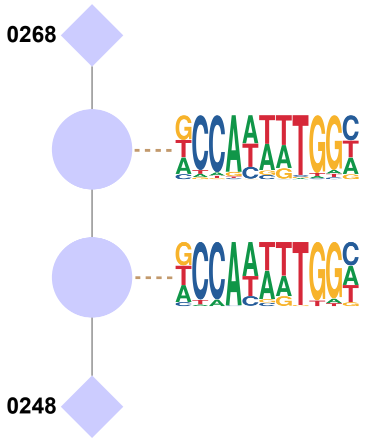

Module 19

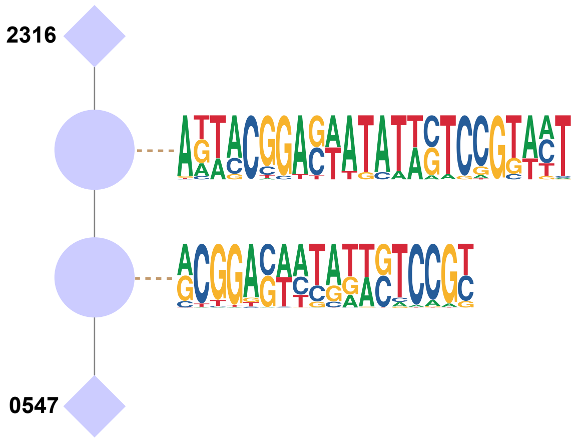

Module 20

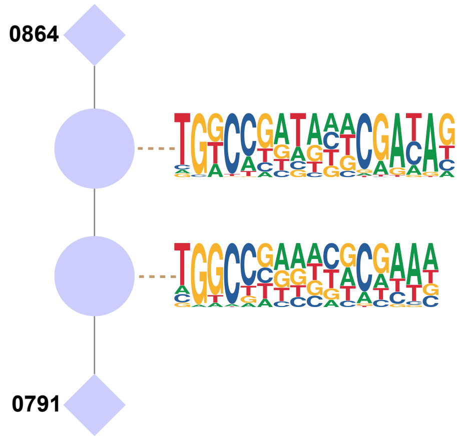

Module 21

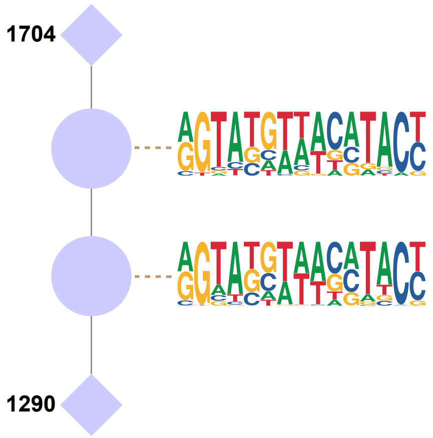

Module 22

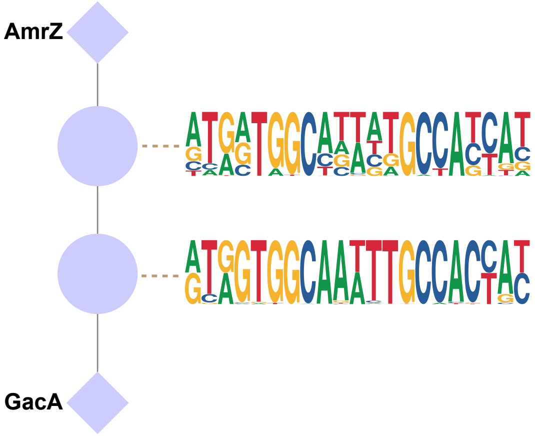

Module 23

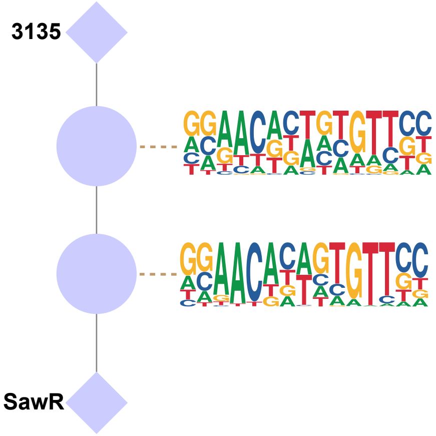

Module 24

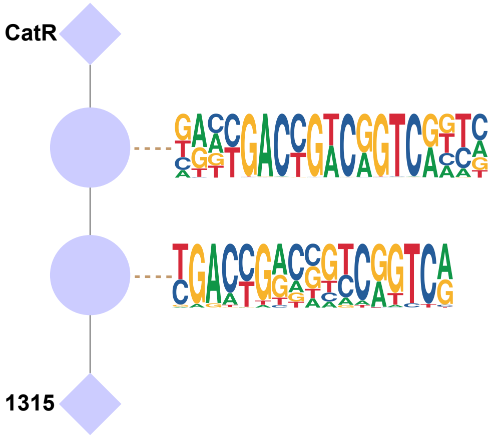

Module 25

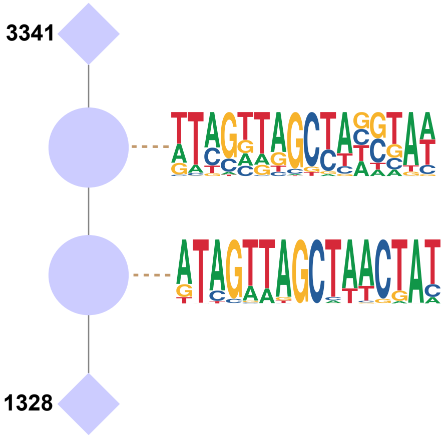

Module 26

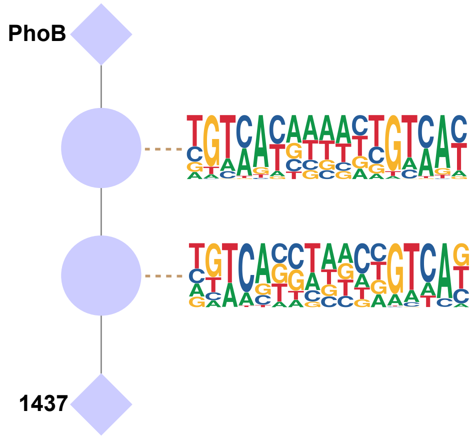

Module 27

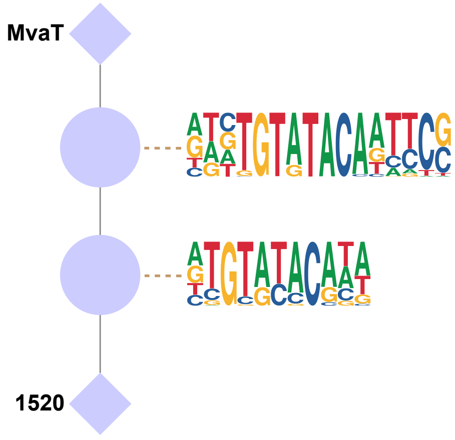

Module 28

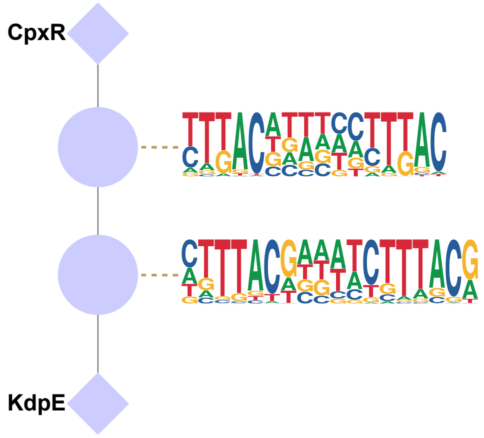

Module 29

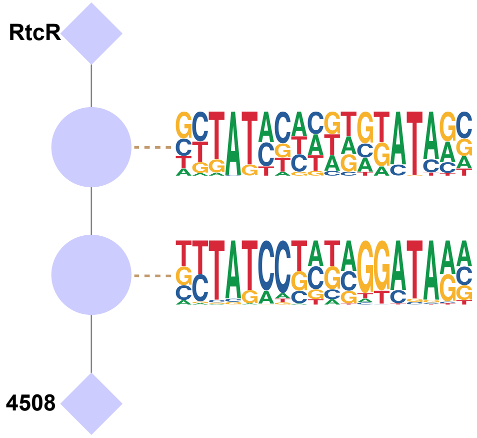

Module 30

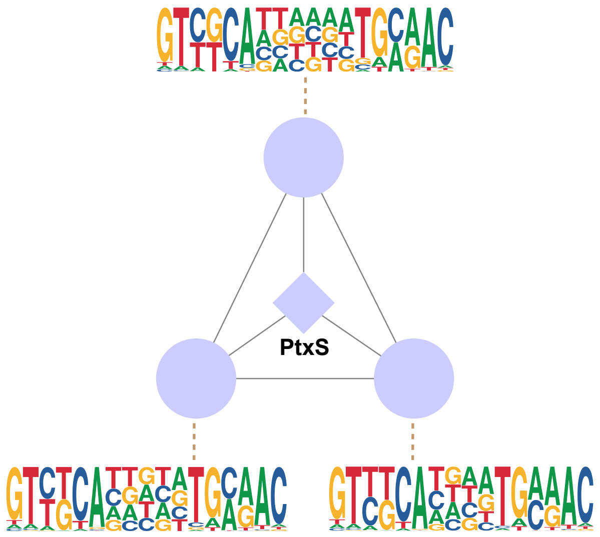

Module 31

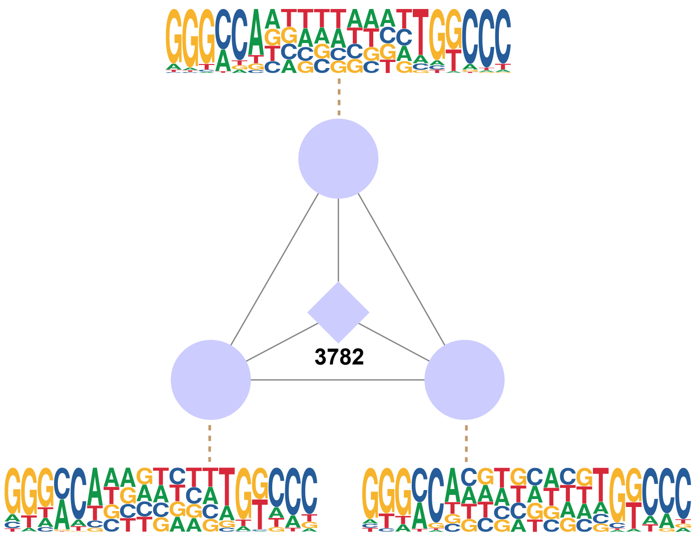

Module 32

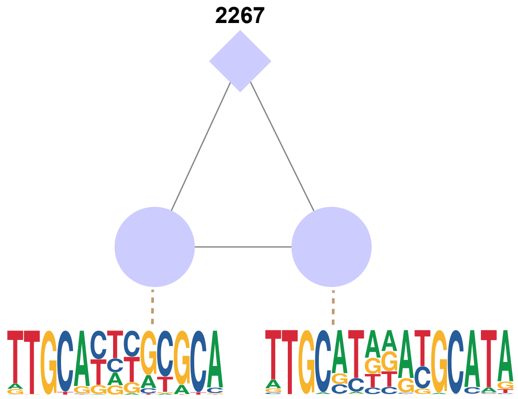

Module 33

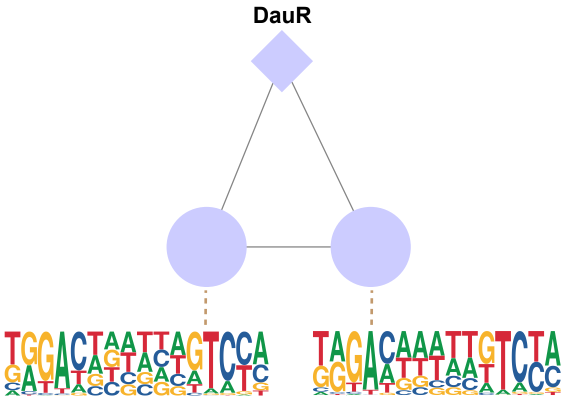

Module 34

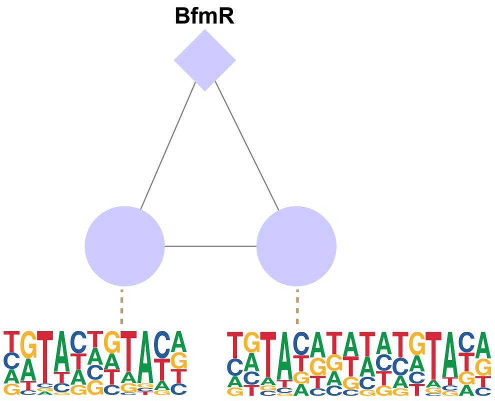

Module 35

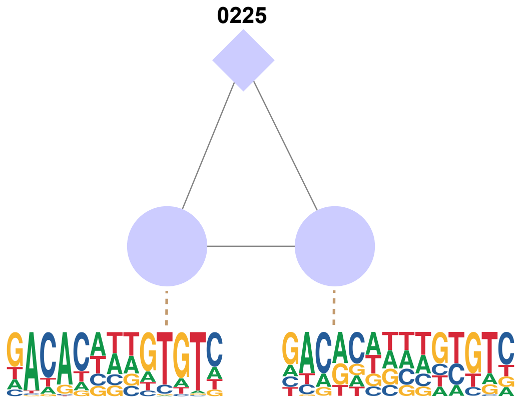

Module 36

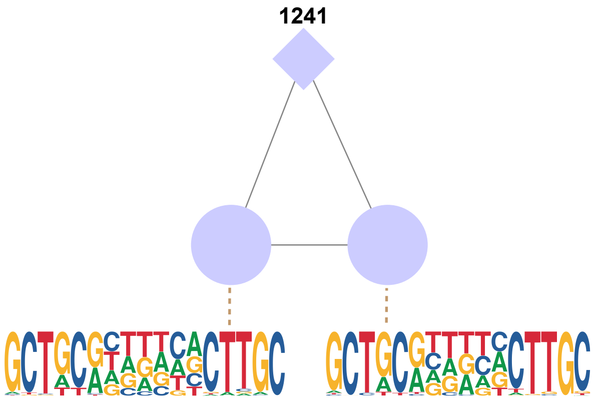

Module 37

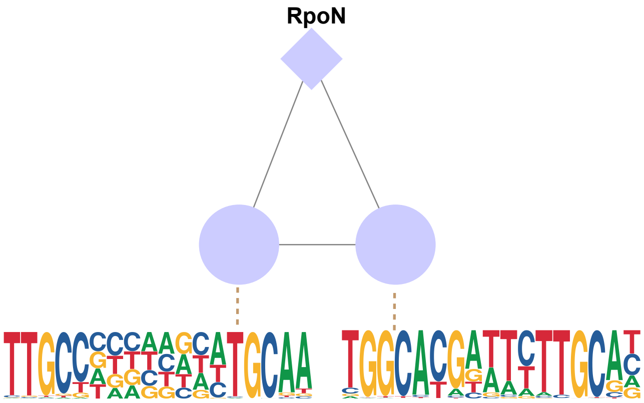

Module 38

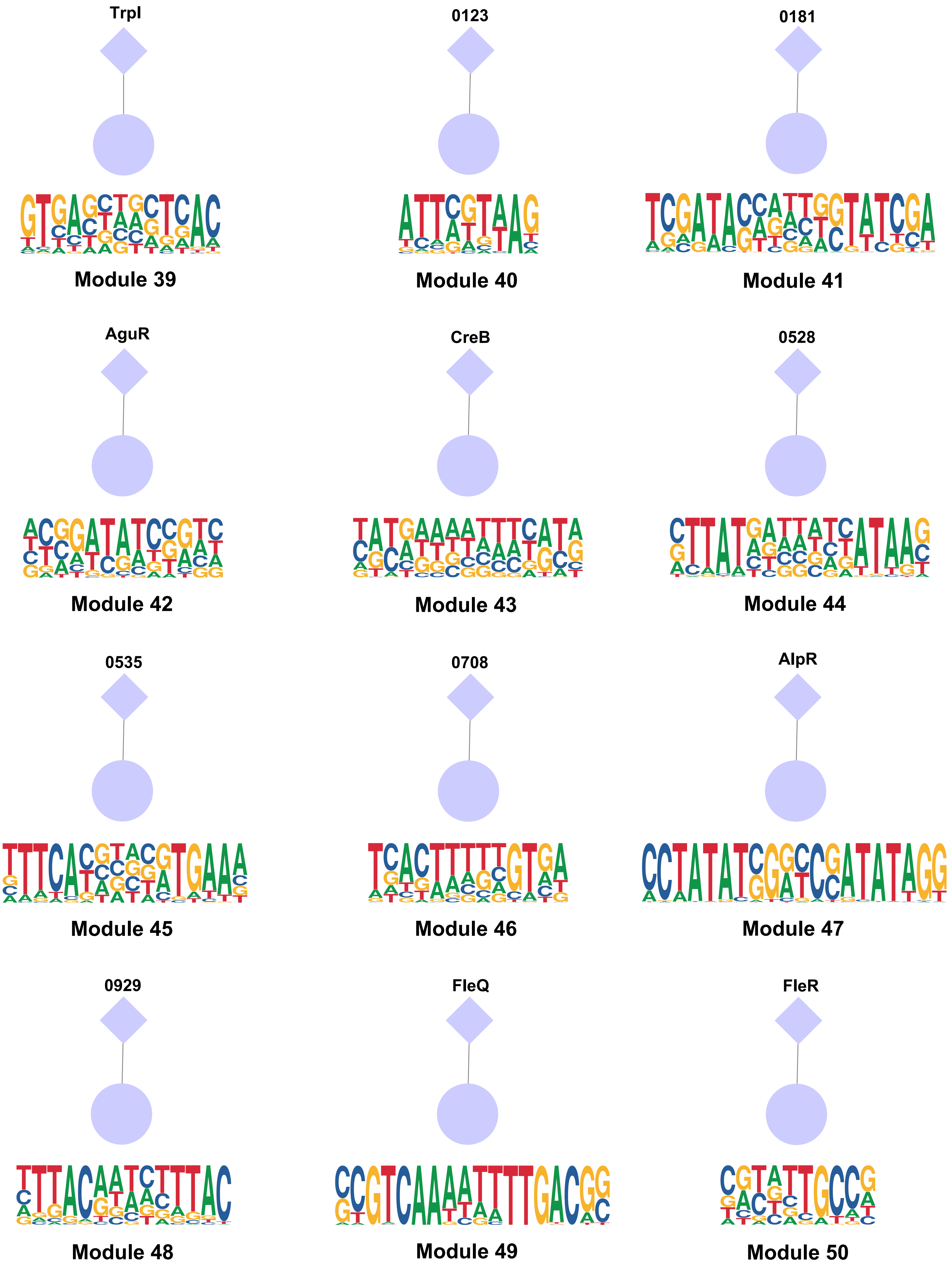
TrpI
Module 39
0123
Module 40
0181
Module 41
AguR
Module 42
CreB
Module 43
0528
Module 44
0535
Module 45
0708
Module 46
AlpR
Module 47
0929
Module 48
FleQ
Module 49
FleR
Module 50

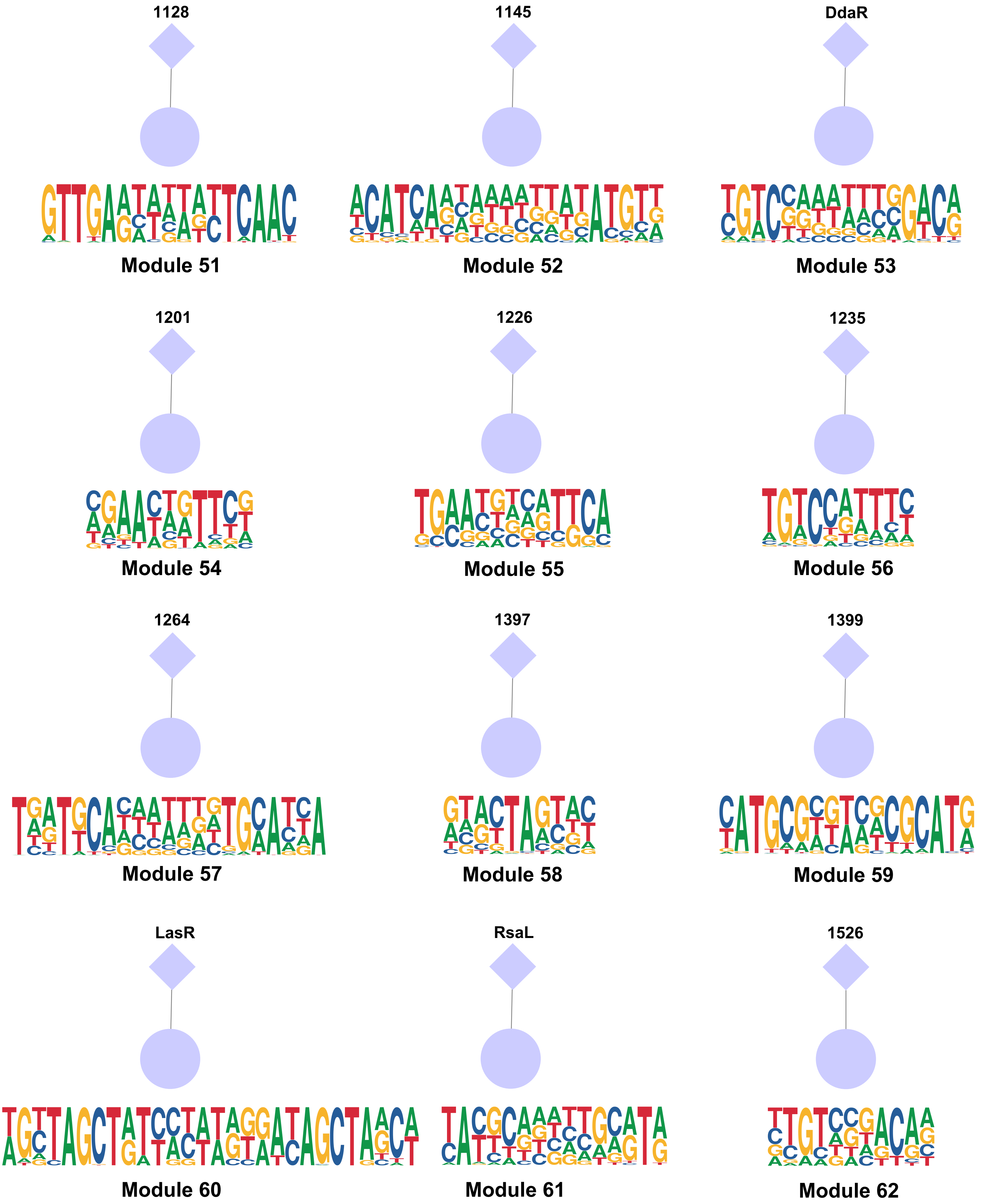
1128
Module 51
1145
Module 52
DdaR
Module 53
1201
Module 54
1226
Module 55
1235
Module 56
1264
Module 57
1397
Module 58
1399
Module 59
LasR
Module 60
RsaL
Module 61
1526
Module 62

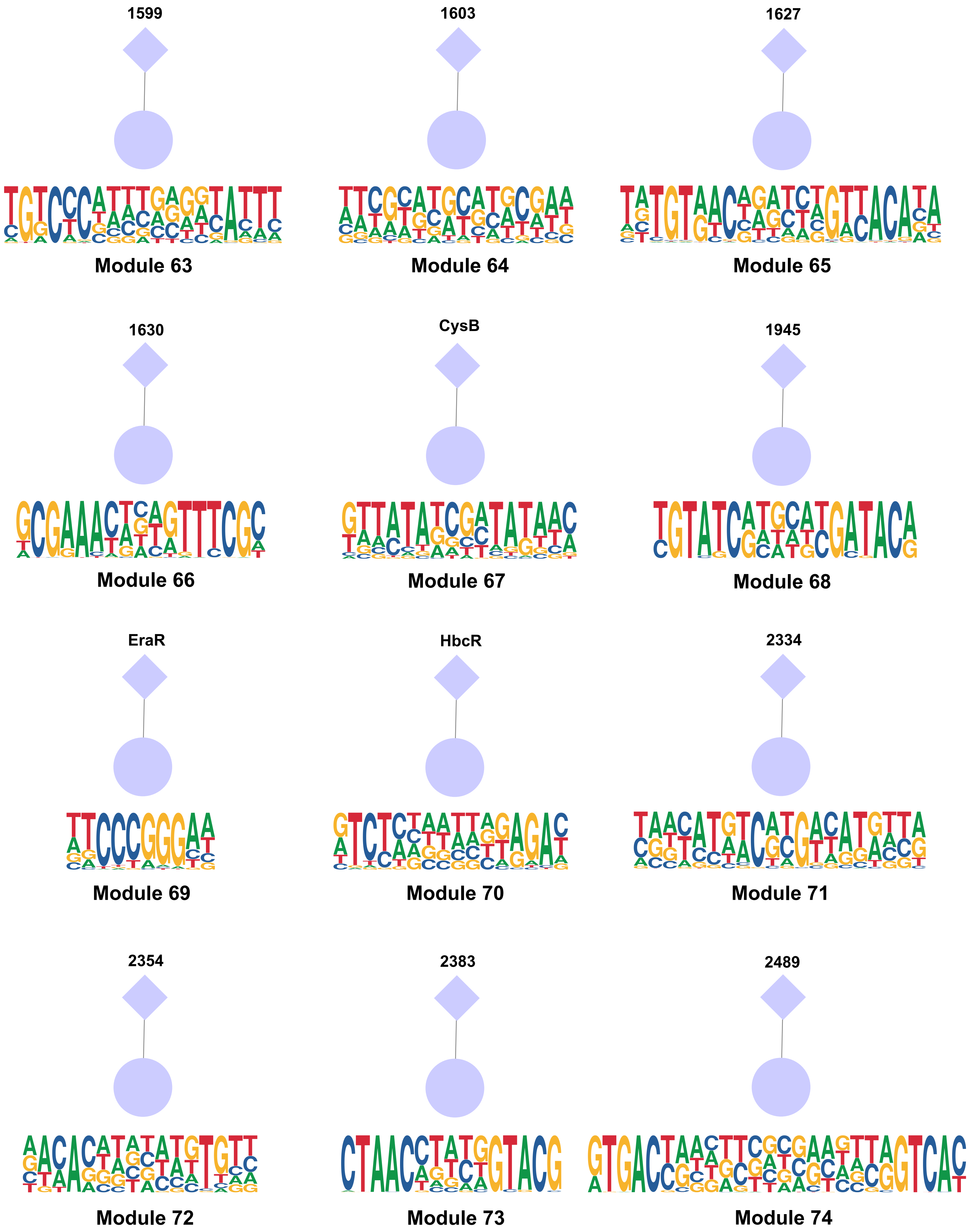

1599
Module 63
1603
Module 64
1627
Module 65
1630
Module 66
CysB
Module 67
1945
Module 68
EraR
Module 69
HbcR
Module 70
2334
Module 71
2354
Module 72
2383
Module 73
2489
Module 74

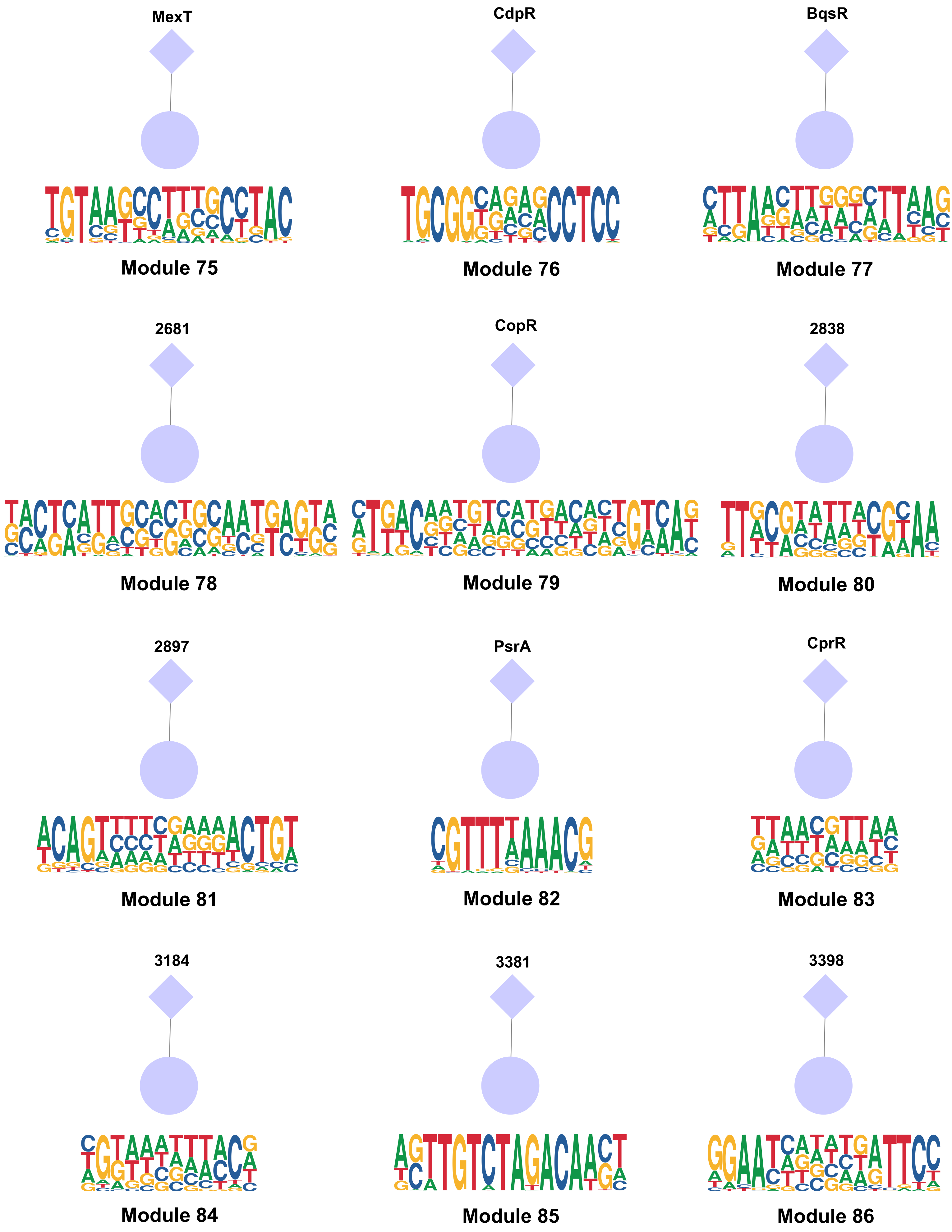
MexT
Module 75
CdpR
Module 76
BqsR
Module 77
2681
Module 78
CopR
Module 79
2838
Module 80
2897
Module 81
PsrA
Module 82
CprR
Module 83
3184
Module 84
3381
Module 85
3398
Module 86

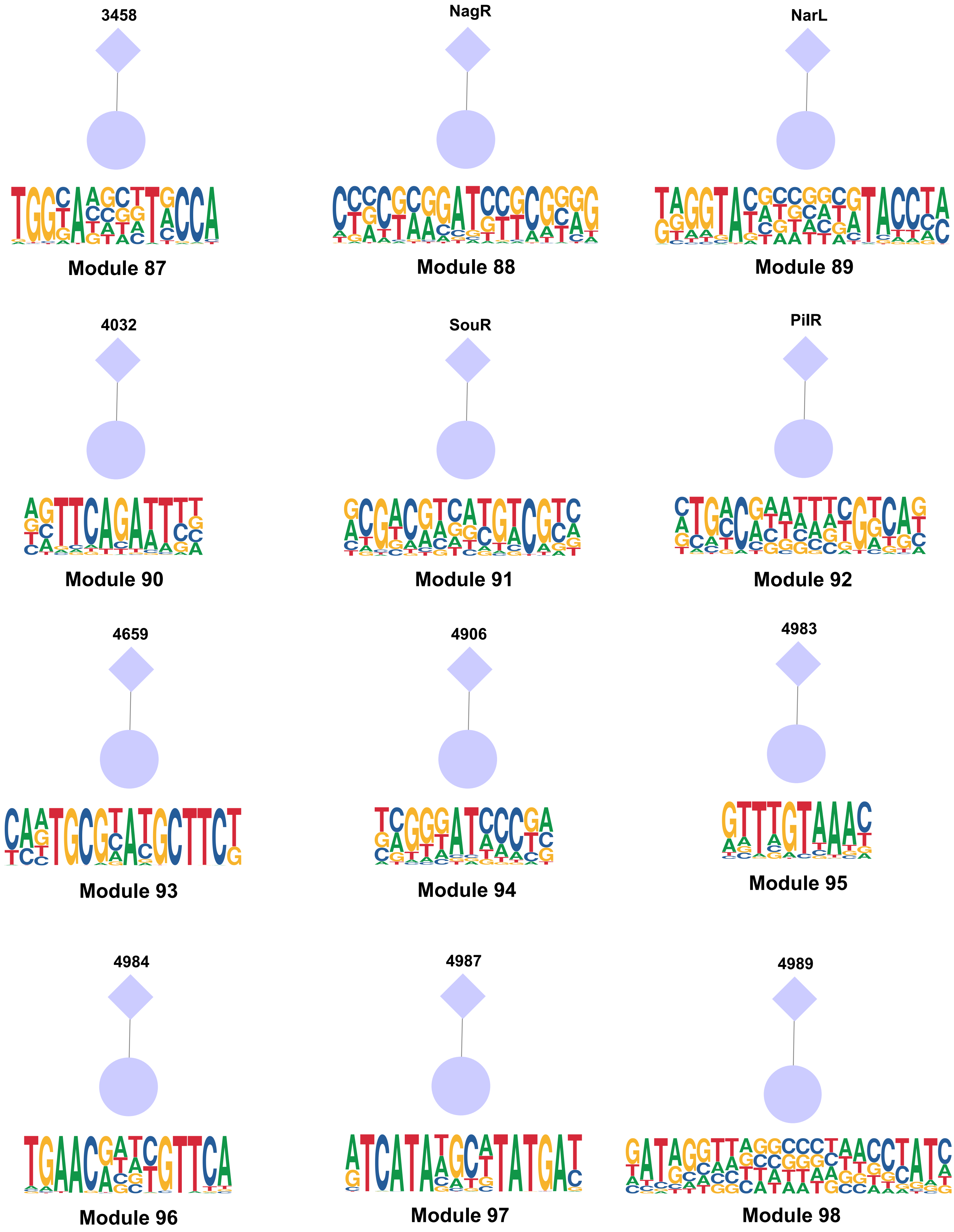
3458
Module 87
NagR
Module 88
NarL
Module 89
4032
Module 90
SouR
Module 91
PilR
Module 92
4659
Module 93
4906
Module 94
4983
Module 95
4984
Module 96
4987
Module 97
4989
Module 98

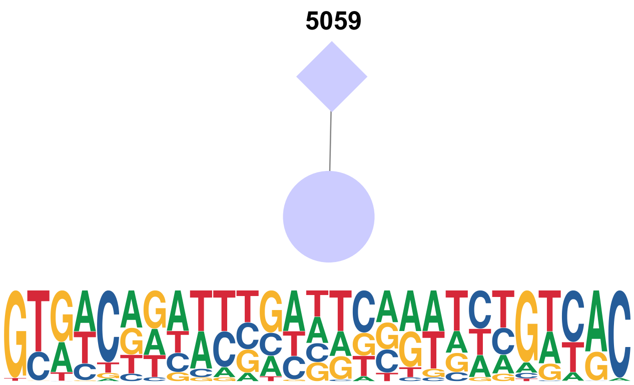

Module 99

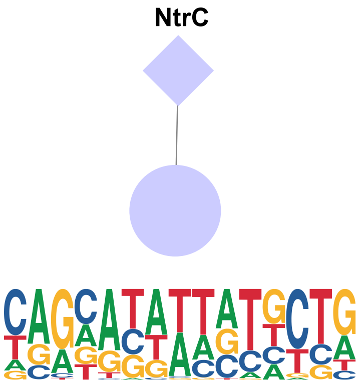

Module 100

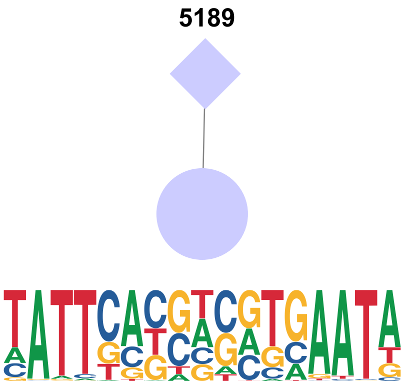

Module 101

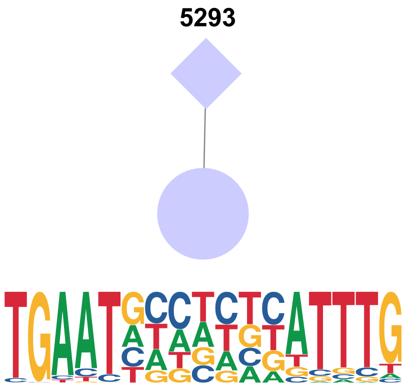

Module 102

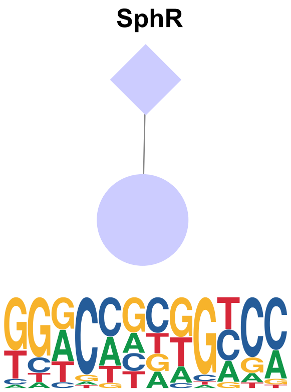

Module 103

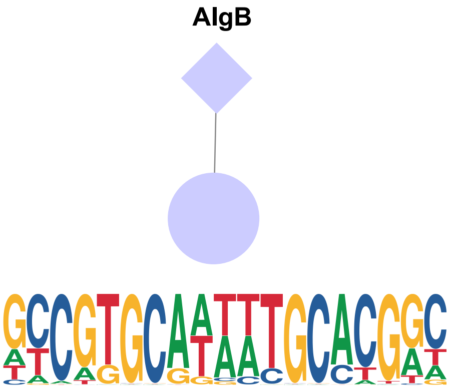

Module 104
